# Supplementary material for: Kir2.1-mediated membrane potential promotes nutrient acquisition and inflammation through regulation of nutrient transporters
Source: Nat Commun. 2022 Jun 21;13:3544. doi: 10.1038/s41467-022-31149-y (PMC9213538; doi:10.1038/s41467-022-31149-y)
Supplement: Supplementary file 1 — Supplementary Information [file 41467_2022_31149_MOESM1_ESM.pdf]

Kir2.1-mediated membrane potential is a critical determinant of nutrient acquisition feeding inflammation

Supplementary information

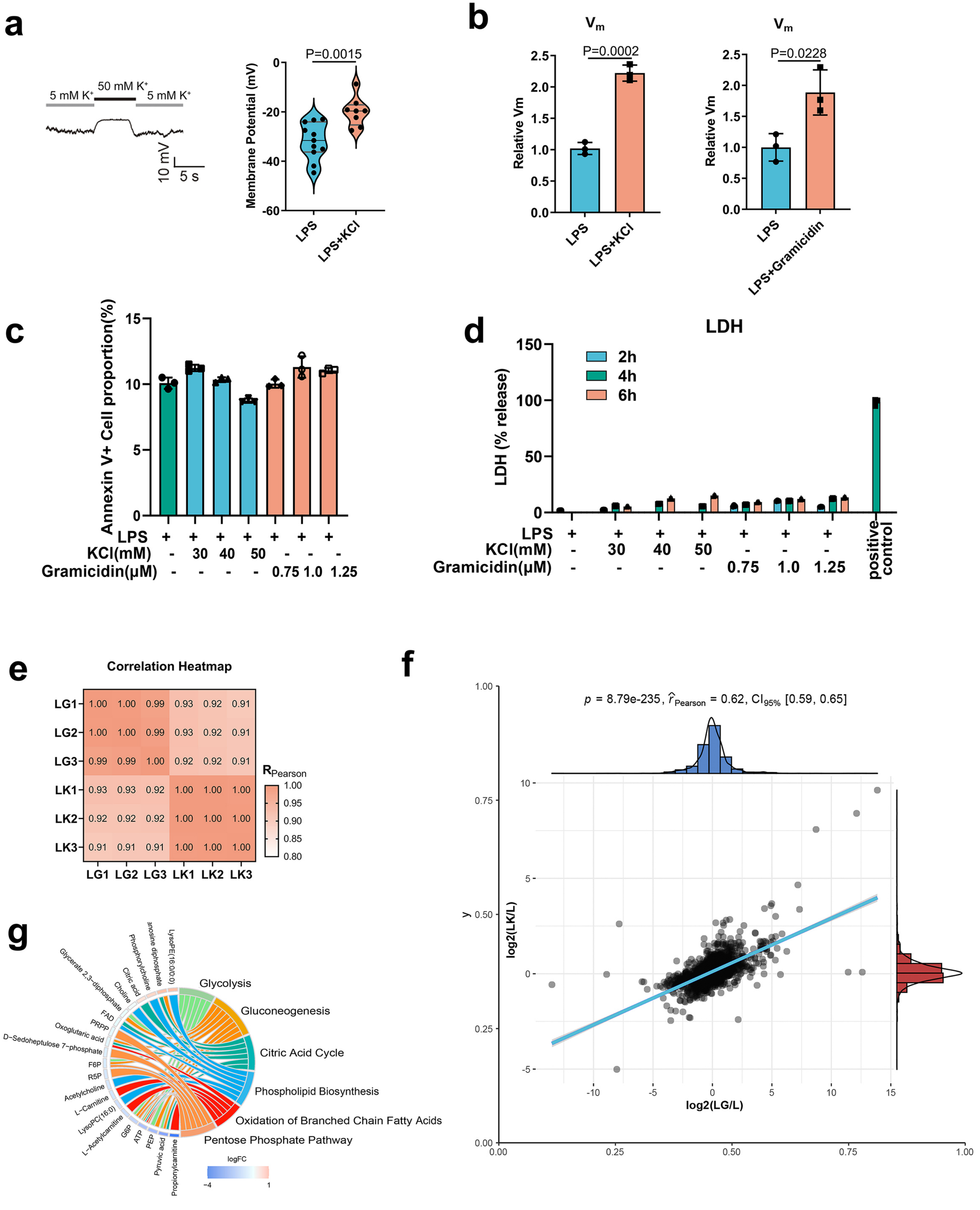
a
5 mM K+
50 mM K+
5 mM K+
10 mV
5 s
P=0.0015
Membrane Potential (mV)
LPS
LPS+KCl
b
Vm
P=0.0002
P=0.0228
Relative Vm
LPS
LPS+KCl
LPS+Gramicidin
c
Annexin V+ Cell proportion(%)
LPS
KCl(mM)
Gramicidin(μM)
d
LDH
LDH (% release)
2h
4h
6h
positive control
e
Correlation Heatmap
LG1
LG2
LG3
LK1
LK2
LK3
RPearson
f
p = 8.79e-235, r̂Pearson = 0.62, CI95% [0.59, 0.65]
log2(LK/L)
log2(LG/L)
g
Glycolysis
Gluconeogenesis
Citric Acid Cycle
Phospholipid Biosynthesis
Oxidation of Branched Chain Fatty Acids
Pentose Phosphate Pathway
logFC

Supplementary Figure 1. Macrophage V_m is essential for nutrient acquisition to fuel inflammation

(a and b) Vm measurement by patch clamp (a) and the Vm-sensitive fluorochrome DiBAC4(3) (b) (a, n=11 and 8; b, n=3; mean ± SD).

(c) Apoptosis analysis of mouse peritoneal macrophages treated with 500 ng/ml LPS in the presence or absence of different concentrations of elevated $[K^+]_e$ and gramicidin for 6 h followed by annexin V and PI staining (n=3, mean ± SD). Representative of three independent experiments.

(d) LDH in the culture medium of mouse peritoneal macrophages treated with 500 ng/ml LPS in the presence or absence of different concentrations of elevated $[K^+]_e$ and gramicidin for different times (n=3, mean ± SD). Representative of three independent experiments.

(e) Correlation heatmap of the LK group (50 mM elevated $[K^+]_e$) and the LG group (LPS plus gramicidin) in the transcriptome.

(f) Correlation analysis of the LK (50 mM elevated $[K^+]_e$) and LG groups (LPS plus gramicidin) in the metabolome.

(g) Chord diagram showing pathway and metabolite enrichment analysis in the highlighted cluster with MetaboAnalyst 5.0 based on the SMPDB database.

LM: LPS+ML133; LG: LPS+Gramicidin; LK: LPS+KCl. Two-tailed unpaired Student's t-test. Source data are provided as a Source Data file.

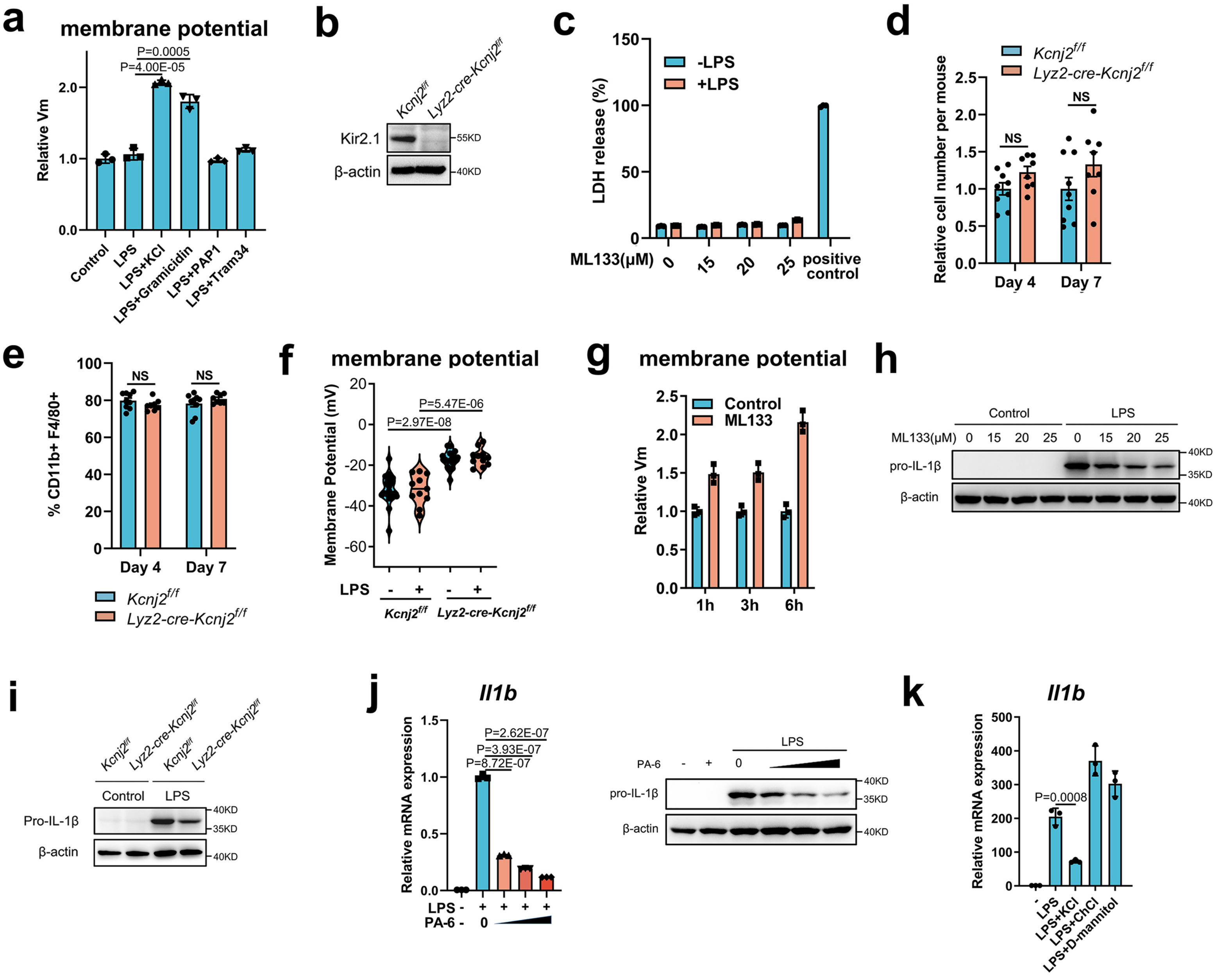
a
membrane potential
P=0.0005
P=4.00E-05
Relative Vm
2.0
1.0
0.0
Control
LPS
LPS+KCl
LPS+Gramicidin
LPS+PAP1
LPS+Tram34
b
Kcnj2f/f
Lyz2-cre-Kcnj2f/f
Kir2.1
β-actin
55KD
40KD
c
LDH release (%)
150
100
50
0
-LPS
+LPS
ML133(μM)
0
15
20
25
positive control
d
Kcnj2f/f
Lyz2-cre-Kcnj2f/f
Relative cell number per mouse
2.5
2.0
1.5
1.0
0.5
0.0
NS
NS
Day 4
Day 7
e
% CD11b+ F4/80+
100
80
60
40
20
0
NS
NS
Day 4
Day 7
Kcnj2f/f
Lyz2-cre-Kcnj2f/f
f
membrane potential
Membrane Potential (mV)
20
0
-20
-40
-60
P=5.47E-06
P=2.97E-08
LPS
-
+
-
+
Kcnj2f/f
Lyz2-cre-Kcnj2f/f
g
membrane potential
Control
ML133
Relative Vm
2.5
2.0
1.5
1.0
0.5
0.0
1h
3h
6h
h
Control
LPS
ML133(μM) 0 15 20 25 0 15 20 25
pro-IL-1β
β-actin
40KD
35KD
40KD
i
Kcnj2f/f
Lyz2-cre-Kcnj2f/f
Kcnj2f/f
Lyz2-cre-Kcnj2f/f
Control
LPS
Pro-IL-1β
β-actin
40KD
35KD
40KD
j
Il1b
Relative mRNA expression
1.5
1.0
0.5
0.0
P=2.62E-07
P=3.93E-07
P=8.72E-07
LPS - + + + +
PA-6 - 0
LPS
PA-6 - + 0
pro-IL-1β
β-actin
40KD
35KD
40KD
k
Il1b
Relative mRNA expression
500
400
300
200
100
0
P=0.0008
-
LPS
LPS+KCl
LPS+ChCl
LPS+D-mannitol

Supplementary Figure 2. Kir2.1 is a critical regulator of macrophage V_m driving inflammation

(a) Vm assessment of mouse peritoneal macrophages with the Vm-sensitive fluorochrome DiBAC4(3). Macrophages were treated with or without 500 ng/ml LPS for 1 h in the presence or absence of elevated $[K^+]_e$ (50 mM), gramicidin (1.25 μM), PAP1 (1 μM), or Tram34 (1 μM) (n=3, mean ± SD). Representative of three independent experiments.

(b) Western blot analysis of Kir2.1 in control and *Lyz2-cre-Kcnj2^f/f^* mouse peritoneal macrophages.

(c) LDH in the culture medium of mouse peritoneal macrophages treated with 500 ng/ml LPS in the presence or absence of different concentrations of ML133 for 6 h (n=3, mean ± SD). Representative of three independent experiments.

(d and e) Percentages and numbers of BMDMs on days 4 and 7 after *ex vivo* differentiation from bone marrow cells from control and *Lyz2-cre-Kcnj2$^{f/f}$* mice. For each mouse, bone marrow cells were flushed from tibias and femurs with cold DMEM, then cultured in 6-well plate with 1/10 of the cells in each well (2 wells were used for further detection in day 4 and day 7) in complete DMEM medium supplemented 10 ng/ml macrophage colony-stimulating factor (PeproTech) to generate BMDMs. In day 4 and 7, cells were harvested and the number and percentage of macrophages ($CD11b^+$ $F4/80^+$) were analyzed by FACS (data are normalized as relative cell number/mouse (control, n = 9; *Lyz2-cre-Kcnj2$^{f/f}$*, n = 8; mean ± SEM). The gating strategy for FACS were shown in supplementary figure 7.

(f) Statistics Vm recorded at 30 s after initiation of recording (*Kcnj2$^{f/f}$*, n = 20 and 11, respectively; *Lyz2-cre-Kcnj2$^{f/f}$*, n = 16 and 11, respectively; mean ± SEM)

(g) Vm assessment using the Vm-sensitive fluorochrome DiBAC4(3) in mouse peritoneal macrophages treated with or without 500 ng/ml LPS for 1, 3, or 6 h in the presence or absence of 25 μM ML133 (n=3, mean ± SD). Representative of three independent experiments.

(h) Western blots of pro-IL-1β expression in mouse peritoneal macrophages treated with 500 ng/ml LPS in the presence or absence of 25 μM ML133 for 6 h.

(i) Western blots of pro-IL-1β expression in lysed mouse peritoneal macrophages from control and Lyz2-cre-*Kcnj2$^{f/f}$* mice treated with 500 ng/ml LPS for 6 h.

(j) qPCR of IL-1β mRNA transcription and Western blots of pro-IL-1β expression in mouse peritoneal macrophages treated with 500 ng/ml LPS for 6 h in the presence or absence of PA-6. (n=3, mean ± SD).

(k) qPCR analysis of IL-1β mRNA transcription levels of mouse peritoneal macrophages treated with 500 ng/ml LPS in the presence of elevated $[K^+]_e$ (50 mM), choline chloride (50 mM), or D-mannitol (50 mM) for 6 h (n=3,

mean ± SD).
Two-tailed unpaired Student's t-test. The qPCR and western blot data are representative of three independent experiments. Source data are provided as a Source Data file.

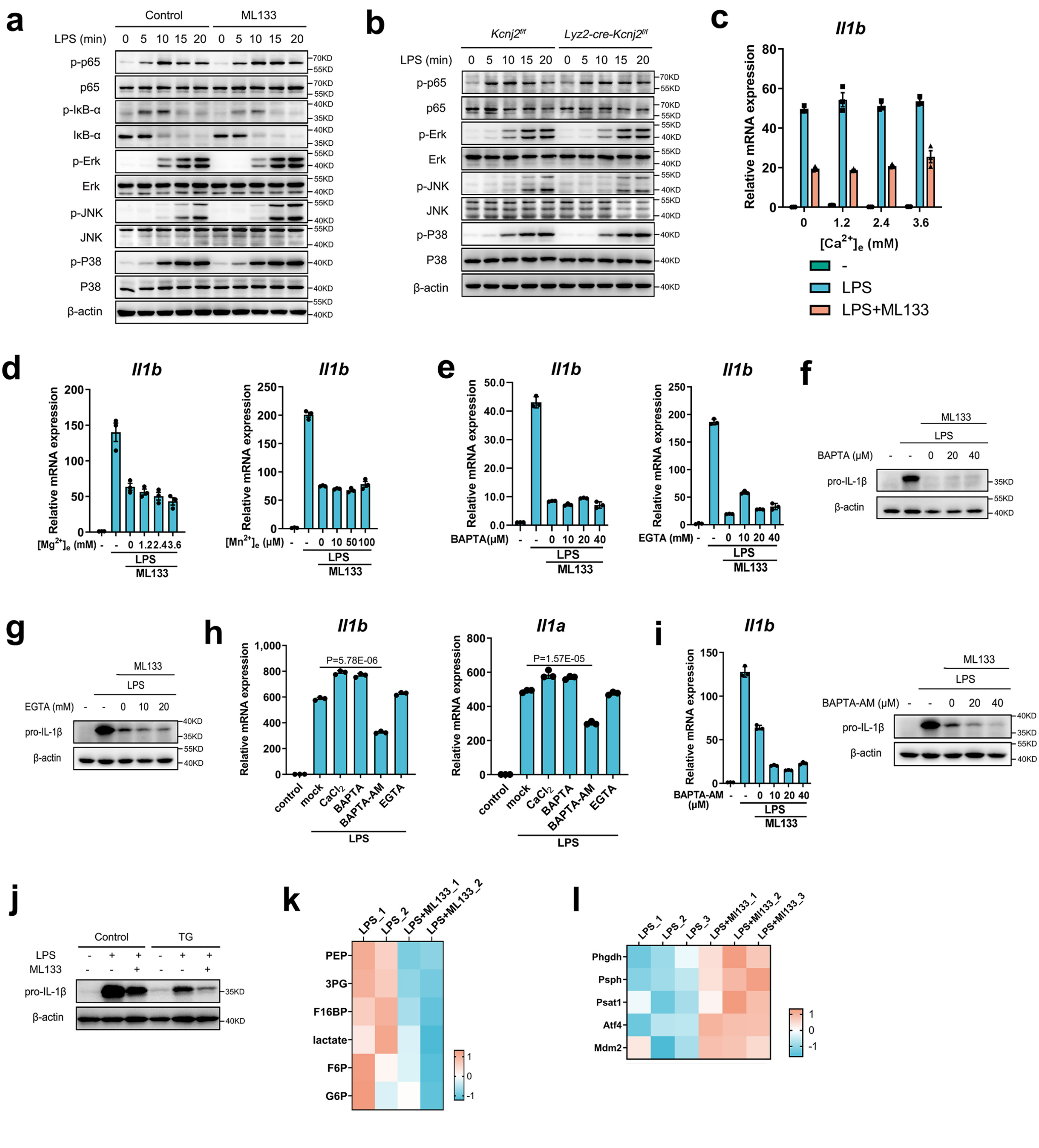

m

GCN2	Metabolic-stress sensing protein that responds to amino acid deprivation by binding uncharged transfer RNAs
PERK	Metabolic-stress sensing protein kinase that responds to low amino acid availability by phosphorylating eIF2a
IMPACT	Translational regulator that ensures constant high levels of translation upon a variety of stress conditions, such as amino acid starvation and glucose deprivation.
SLC38A2	Sodium-dependent amino acid transporter that responds to amino acid starvation by GCN pathway.

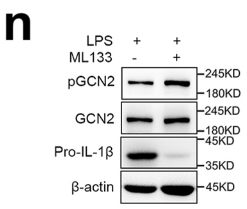

Supplementary Figure 3. Kir2.1-mediated V_m endows macrophages with the metabolic signature of inflammation

(a and b) Activation of TLR4-mediated downstream signaling pathways in 500 ng/ml LPS-treated peritoneal macrophages in the presence or absence of ML133 (25 μM).

(c and d) qPCR analysis of IL-1β mRNA transcription levels in mouse peritoneal macrophages treated with 500 ng/ml LPS and 25 μM ML133 in the presence of different concentrations of extracellular Ca^{2+} (c), Mg^{2+} and Mn^{2+} (d) for 6 h (c, n=3; d, n=3; mean ± SD).

(e to i) IL-1β mRNA (e, h, and i), IL-1α and TNF-α (h) transcription, and pro-IL-1β protein expression levels (f, g and i) of mouse peritoneal macrophages stimulated by 500 ng/ml LPS with 25 μM ML133 in the presence of BAPTA (e and f), EGTA (e and g), and BAPTA-AM (i) for 6 h followed. (h) qPCR analysis of macrophages stimulated by 500 ng/ml LPS in the presence of $CaCl_2$ (2 mM), BAPTA (20 μM), BAPTA-AM (20 μM), and EGTA (10 mM) for 6 h. (e, n=3; h, n=3; i, n=3; mean ± SD).

(j) Western blots of pro-IL-1β protein expression levels of mouse peritoneal macrophages treated with 500 ng/ml LPS and 25 μM ML133 in the presence or absence of 1 μM thapsigargin (TG) as indicated for 6 h.

(k) Metabolites of glycolysis identified in targeted metabolomics. Mouse peritoneal macrophages were treated with 500 ng/ml LPS in the presence or absence of ML133 (25 μM) for 6 h, then cell metabolites were extracted and analyzed by LC-MS (n = 2).

(l) Heatmap of RNA-seq analysis of enzyme genes and master regulators for the SSP in BMDMs treated with 500 ng/ml LPS in the presence or absence of ML133 (25 μM) for 6 h (n = 3).

(m) Brief descriptions of the four overlapped genes upregulated by both ML133 (25 μM) and Kir2.1 genetic-deficient macrophages.

(n) Western blot analysis of GCN2 activation of mouse peritoneal macrophages treated with LPS in the presence or absence of ML133 (25 μM) for 6 h.

Two-tailed unpaired Student’s t-test. The qPCR and western blot data are representative of three independent experiments. Source data are provided as a Source Data file.

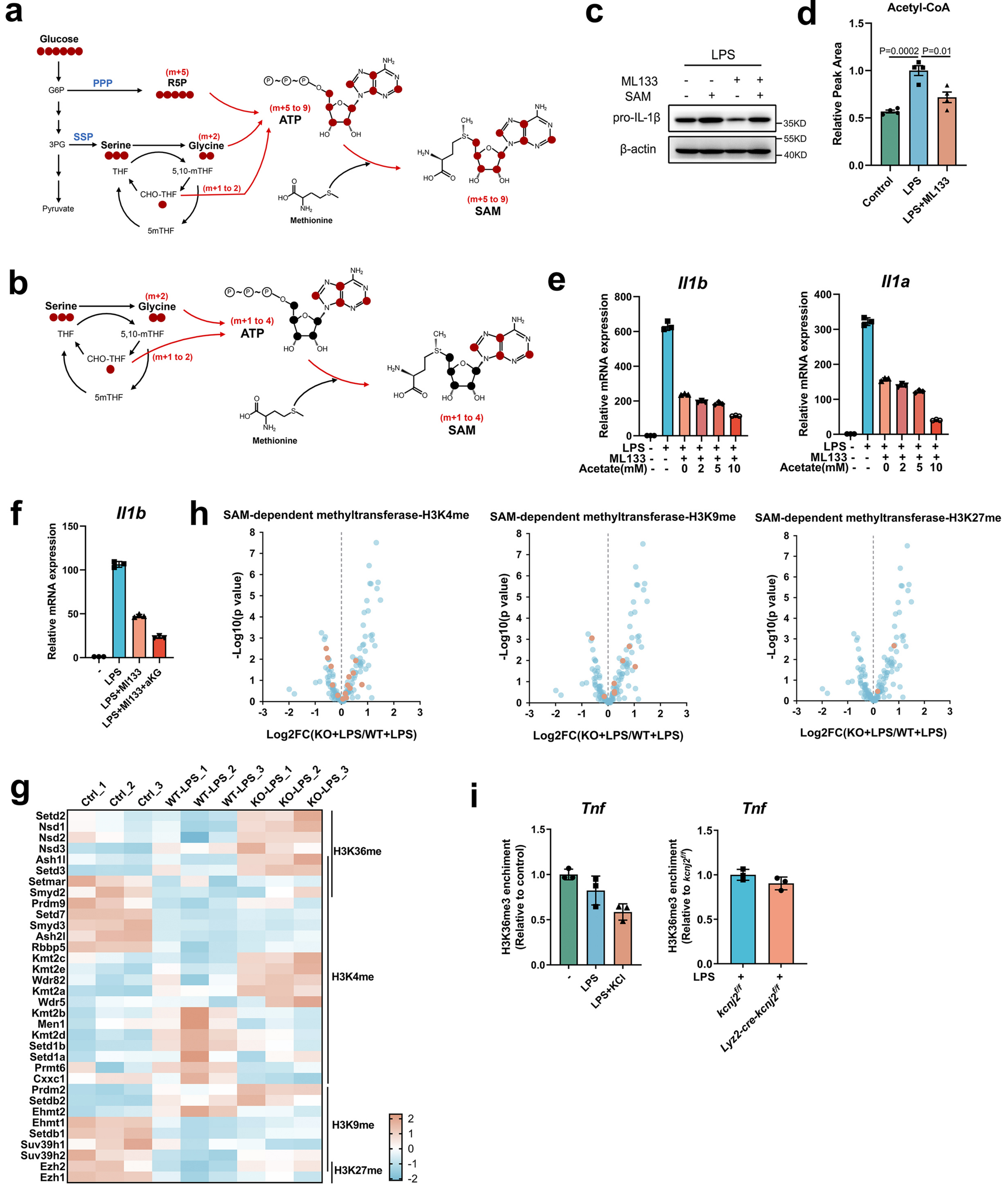
a
Glucose
G6P
PPP
(m+5)
R5P
3PG
SSP
Serine
(m+2)
Glycine
THF
5,10-mTHF
CHO-THF
(m+1 to 2)
5mTHF
Pyruvate
(m+5 to 9)
ATP
Methionine
(m+5 to 9)
SAM
b
Serine
(m+2)
Glycine
THF
5,10-mTHF
CHO-THF
(m+1 to 2)
5mTHF
(m+1 to 4)
ATP
Methionine
(m+1 to 4)
SAM
c
LPS
ML133
SAM
pro-IL-1β
β-actin
35KD
55KD
40KD
d
Acetyl-CoA
Relative Peak Area
P=0.0002
P=0.01
Control
LPS
LPS+ML133
e
Il1b
Il1a
Relative mRNA expression
LPS
ML133
Acetate(mM)
f
Il1b
Relative mRNA expression
LPS
LPS+Ml133
LPS+Ml133+aKG
h
SAM-dependent methyltransferase-H3K4me
SAM-dependent methyltransferase-H3K9me
SAM-dependent methyltransferase-H3K27me
-Log10(p value)
Log2FC(KO+LPS/WT+LPS)
g
Ctrl_1
Ctrl_2
Ctrl_3
WT-LPS_1
WT-LPS_2
WT-LPS_3
KO-LPS_1
KO-LPS_2
KO-LPS_3
Setd2
Nsd1
Nsd2
Nsd3
Ash1l
Setd3
Setmar
Smyd2
Prdm9
Setd7
Smyd3
Ash2l
Rbbp5
Kmt2c
Kmt2e
Wdr82
Kmt2a
Wdr5
Kmt2b
Men1
Kmt2d
Setd1b
Setd1a
Prmt6
Cxxc1
Prdm2
Setdb2
Ehmt2
Ehmt1
Setdb1
Suv39h1
Suv39h2
Ezh2
Ezh1
H3K36me
H3K4me
H3K9me
H3K27me
i
Tnf
Tnf
H3K36me3 enchiment (Relative to control)
H3K36me3 enchiment (Relative to kcnj2f/f)
LPS
LPS+KCl
kcnj2f/f
Lyz2-cre-kcnj2f/f

Supplementary Figure 4. Kir2.1-mediated V_m supports nutrient uptake for SAM generation to induce a metabolism-responsive inflammatory program

(a and b) Schematic of derivation and contribution of glucose-derived (a) and serine-derived (b) carbon atoms to SAM synthesis.

(c) Western blots of pro-IL-1β protein expression in mouse peritoneal macrophages treated with 500 ng/ml LPS for 6 h in the presence of ML133 (25 μM) with or without SAM.

(d) Relative Acetyl-CoA levels analyzed by unbiased metabolomics. (c, n=4; mean ± SEM).

(e) qPCR analysis of IL-1β and IL-1α mRNA transcription levels in peritoneal macrophages treated with 500 ng/ml LPS for 6 h in the presence or absence of ML133 (25 μM) and different concentrations of acetate (n=3, mean ± SD).

(f) qPCR analysis of IL-1β mRNA transcription levels in peritoneal macrophages treated with 500 ng/ml LPS for 6 h in the presence or absence of ML133 (25 μM) and α-KG (n=3, mean ± SD).

(g and h) Heatmap (g) and Volcano plot (h) analysis of gene expression of SAM-dependent methyltransferase by RNA-seq in BMDMs treated with 500 ng/ml LPS for 6 h. The expression levels of methyltransferase genes (highlighted in h) (Wagner and Carpenter, 2012) among 183 annotated SAM-dependent methyltransferase genes (Kottakis et al., 2016) were analyzed.

(i) ChIP-qPCR analysis of H3K36me3 enrichment in the *Tnf* gene in mouse peritoneal macrophages from control and *Lyz2-cre-Kcnj2*$^{f/f}$ mice treated with 500 ng/ml LPS in the presence or absence of ML133 (25 μM) for 6 h (n=3, mean ± SD).

Two-tailed unpaired Student's t-test. The qPCR and western blot data are representative of three independent experiments. Source data are provided as a Source Data file.

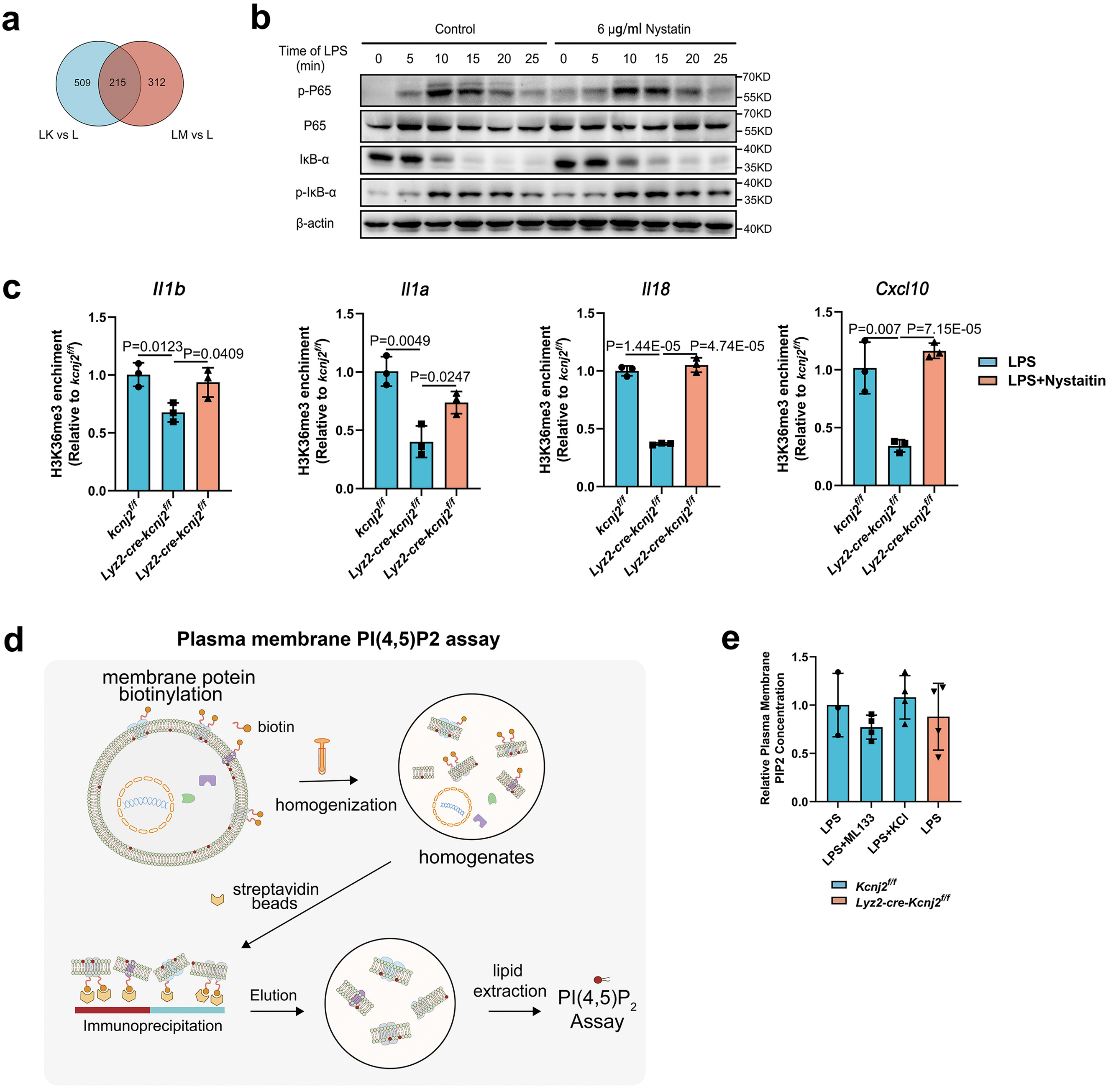
a
509
215
312
LK vs L
LM vs L
b
Control
6 μg/ml Nystatin
Time of LPS (min)
0 5 10 15 20 25 0 5 10 15 20 25
p-P65
P65
IκB-α
p-IκB-α
β-actin
70KD
55KD
40KD
35KD
c
Il1b
Il1a
Il18
Cxcl10
H3K36me3 enchiment (Relative to kcnj2f/f)
P=0.0123
P=0.0409
P=0.0049
P=0.0247
P=1.44E-05
P=4.74E-05
P=0.007
P=7.15E-05
kcnj2f/f
Lyz2-cre-kcnj2f/f
LPS
LPS+Nystaitin
d
Plasma membrane PI(4,5)P2 assay
membrane potein biotinylation
biotin
homogenization
homogenates
streptavidin beads
Immunoprecipitation
Elution
lipid extraction
PI(4,5)P2 Assay
e
Relative Plasma Membrane PIP2 Concentration
LPS
LPS+ML133
LPS+KCl
Kcnj2f/f
Lyz2-cre-Kcnj2f/f

Supplementary Figure 5. Kir2.1-mediated V_m drives nutrient uptake by retaining nutrient transporters on the macrophage cell surface

(a) Venn diagram of overlapped differential membrane protein analysis by MS. LM: LPS+ML133; LK: LPS+KCl.

(b) Activation of TLR4-mediated downstream signaling pathways in peritoneal macrophages treated with 500 ng/ml LPS in the presence or absence of 6 μg/ml Nystatin.

(c) ChIP-qPCR analysis of H3K36me3 enrichment in the *Il1a*, *Il1b*, *Il18*, and *Cxcl10* genes in mouse peritoneal macrophages from control and *Lyz2-cre-Kcnj2$^{f/f}$* mice treated with 500 ng/ml LPS in the presence or absence of 6 μg/ml nystatin for 6 h (n=3, mean ± SD).

(d and e) Schematic diagram of plasma membrane lipid extraction for PI(4,5)P2 ELISA analysis (d) and the relative amount of plasma membrane PI(4,5)P2 (e). (n=3, 4, 4, 4 respectively; mean ± SD).

Two-tailed unpaired Student's t-test. The ChIP-qPCR and western blot data are representative of three independent experiments. Source data are provided as a Source Data file.

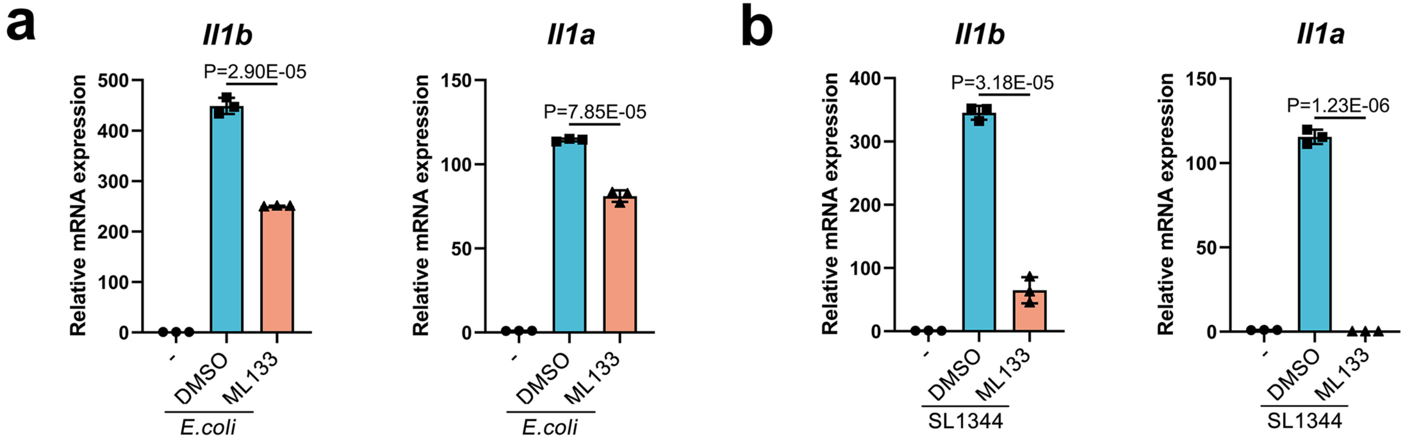

a
Il1b
P=2.90E-05
Relative mRNA expression
500
400
300
200
100
0
-
DMSO
ML133
E.coli
Il1a
P=7.85E-05
Relative mRNA expression
150
100
50
0
-
DMSO
ML133
E.coli
b
Il1b
P=3.18E-05
Relative mRNA expression
400
300
200
100
0
-
DMSO
ML133
SL1344
Il1a
P=1.23E-06
Relative mRNA expression
150
100
50
0
-
DMSO
ML133
SL1344

Supplementary Figure 6. Kir2.1 repression alleviates inflammation triggered by pathogenic or danger signals in mouse models and human samples

(a and b) qPCR analysis of IL-1β and IL-1α mRNA transcription levels in peritoneal macrophages infected with *E. coli* or *salmonella SL1344* for 6 h in the presence or absence of ML133 (25 μM) (n=3, mean ± SD).

Two-tailed unpaired Student's t-test. Representative of three independent experiments. Source data are provided as a Source Data file.

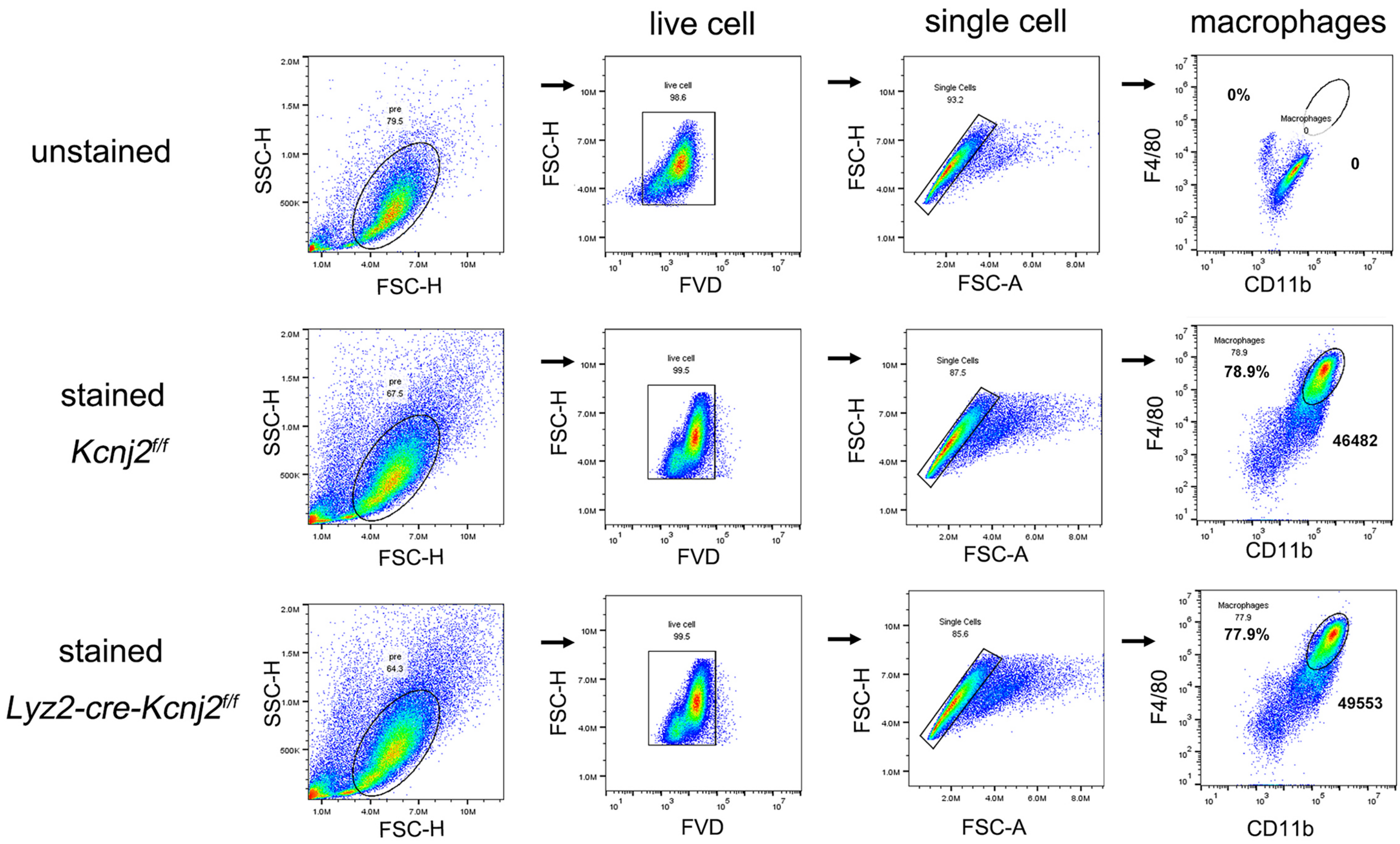

live cell
single cell
macrophages
unstained
stained
Kcnj2f/f
stained
Lyz2-cre-Kcnj2f/f
SSC-H
FSC-H
FVD
FSC-A
F4/80
CD11b
pre
79.5
67.5
64.3
live cell
98.6
99.5
99.5
Single Cells
93.2
87.5
85.6
Macrophages
0
0%
0
78.9
78.9%
46482
77.9
77.9%
49553

Supplementary Figure 7. Gating strategy for FACS in supplemental figure 2d and 2e.

Shown above is the representative gating strategy for FACS of macrophages differentiation in supplementary figure 2d and 2e.

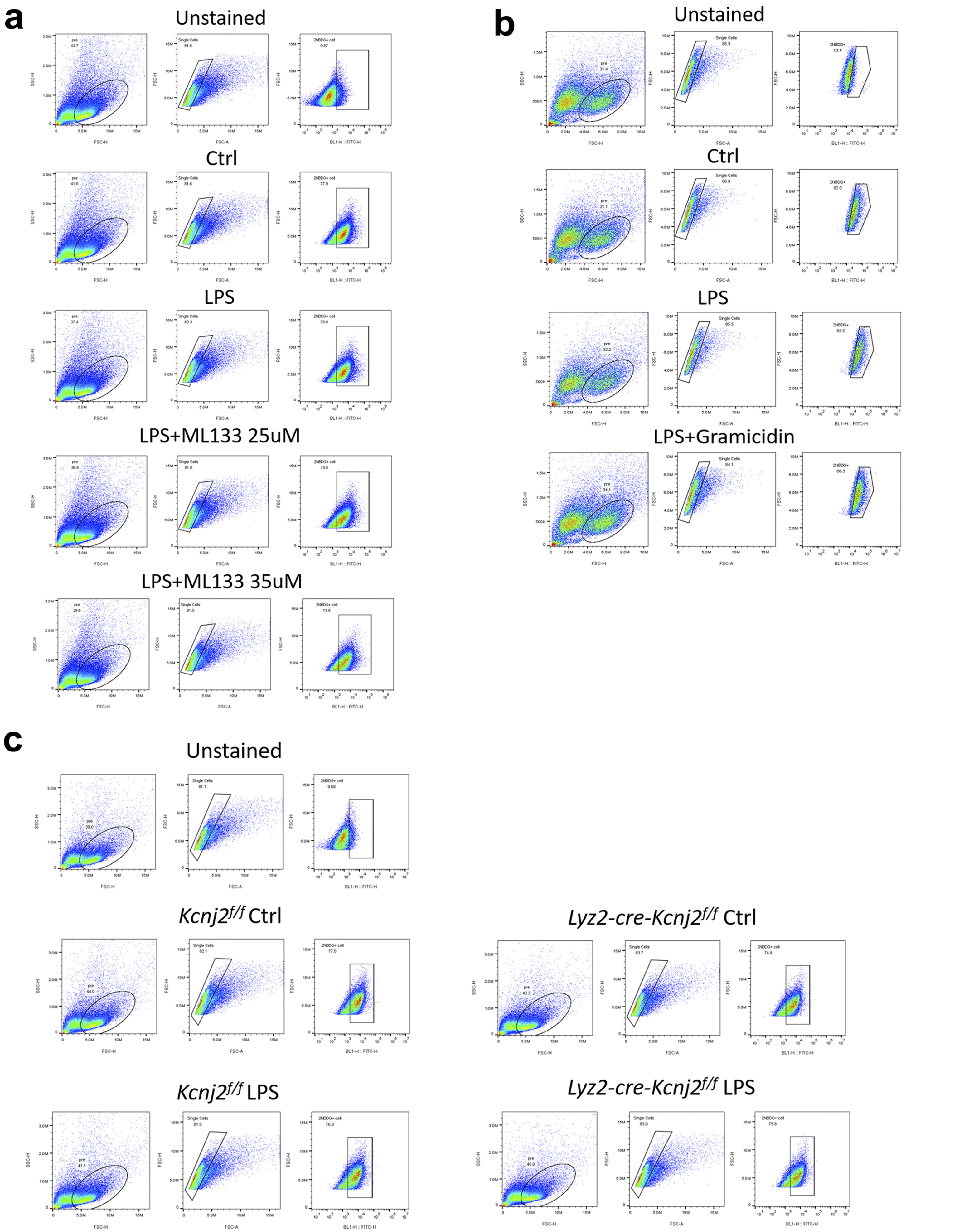
a
Unstained
Ctrl
LPS
LPS+ML133 25uM
LPS+ML133 35uM
b
Unstained
Ctrl
LPS
LPS+Gramicidin
c
Unstained
Kcnj2$^{f/f}$ Ctrl
Lyz2-cre-Kcnj2$^{f/f}$ Ctrl
Kcnj2$^{f/f}$ LPS
Lyz2-cre-Kcnj2$^{f/f}$ LPS

Supplementary Figure 8. Gating strategy for FACS in figure 3f.
Shown above is the representative gating strategy for FACS of *in vitro* glucose uptake in macrophages in figure 3f.

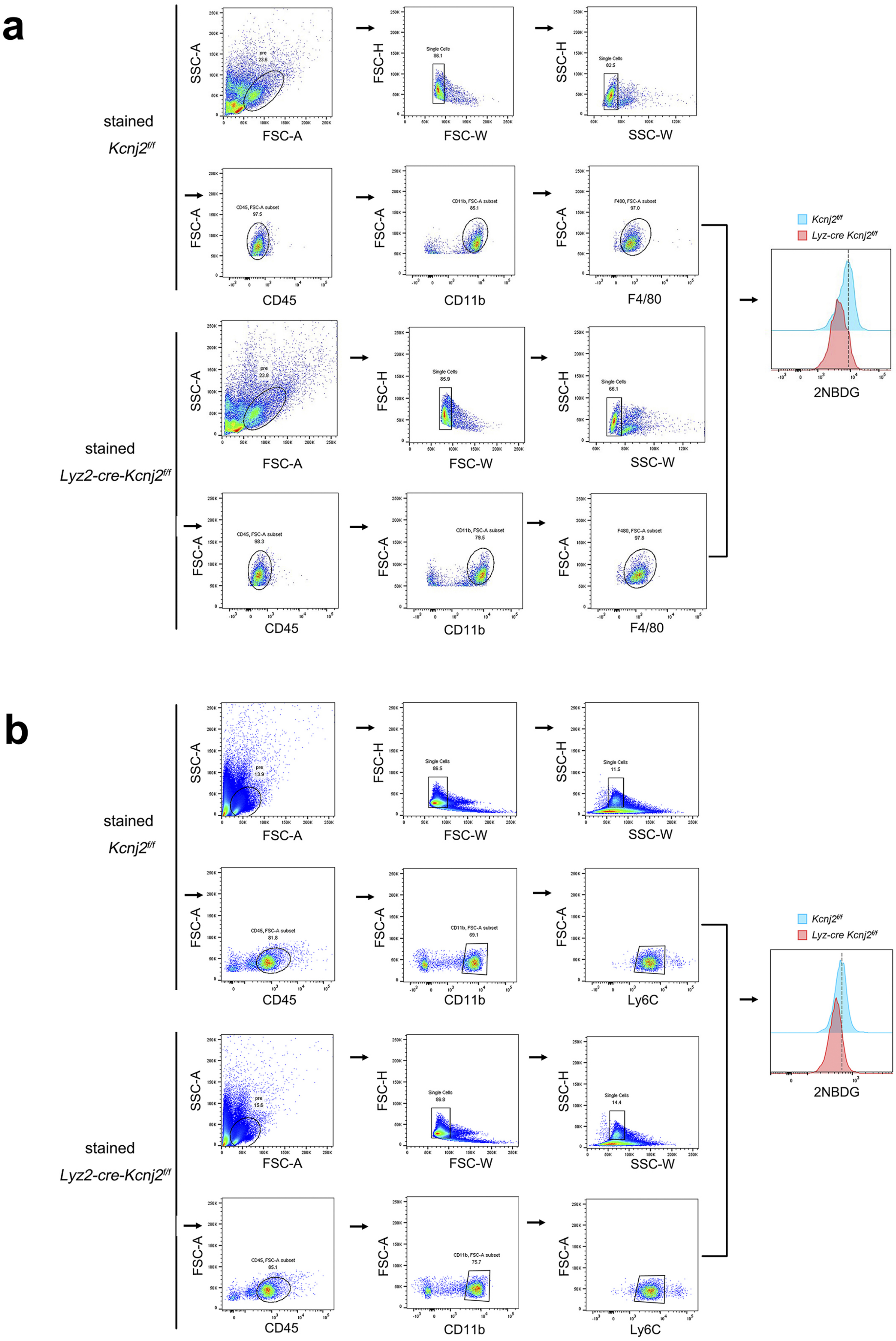
a
stained
Kcnj2f/f
stained
Lyz2-cre-Kcnj2f/f
SSC-A
FSC-A
FSC-H
FSC-W
SSC-H
SSC-W
CD45
CD11b
F4/80
Kcnj2f/f
Lyz-cre Kcnj2f/f
2NBDG
b
stained
Kcnj2f/f
stained
Lyz2-cre-Kcnj2f/f
SSC-A
FSC-A
FSC-H
FSC-W
SSC-H
SSC-W
CD45
CD11b
Ly6C
Kcnj2f/f
Lyz-cre Kcnj2f/f
2NBDG

Supplementary Figure 9. Gating strategy for FACS in figure 3g.
Shown above is the representative gating strategy for FACS of *in vivo* glucose uptake in macrophages in figure 3g. a, gating strategy for macrophages in PEC; b, gating strategy for monocytes in PBMCs.

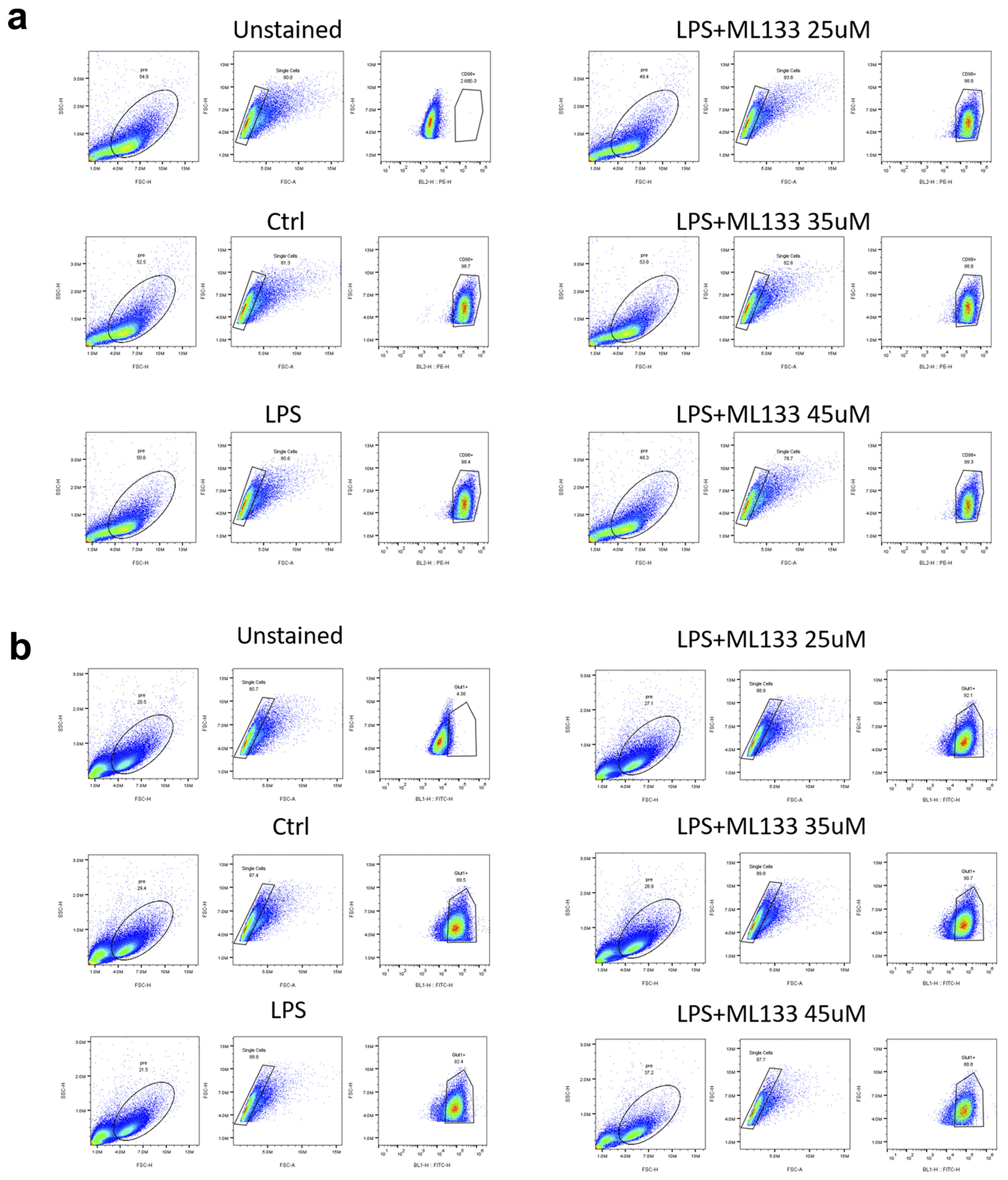
a
Unstained
LPS+ML133 25uM
Ctrl
LPS+ML133 35uM
LPS
LPS+ML133 45uM
b
Unstained
LPS+ML133 25uM
Ctrl
LPS+ML133 35uM
LPS
LPS+ML133 45uM

Supplementary Figure 10. gating strategy for FACS in figure 5f, 5g and 5h.
Shown above is the representative gating strategy for FACS of membrane 4F2hc (a) and Glut1 (b) in macrophages in figure 5f, 5g and 5h.

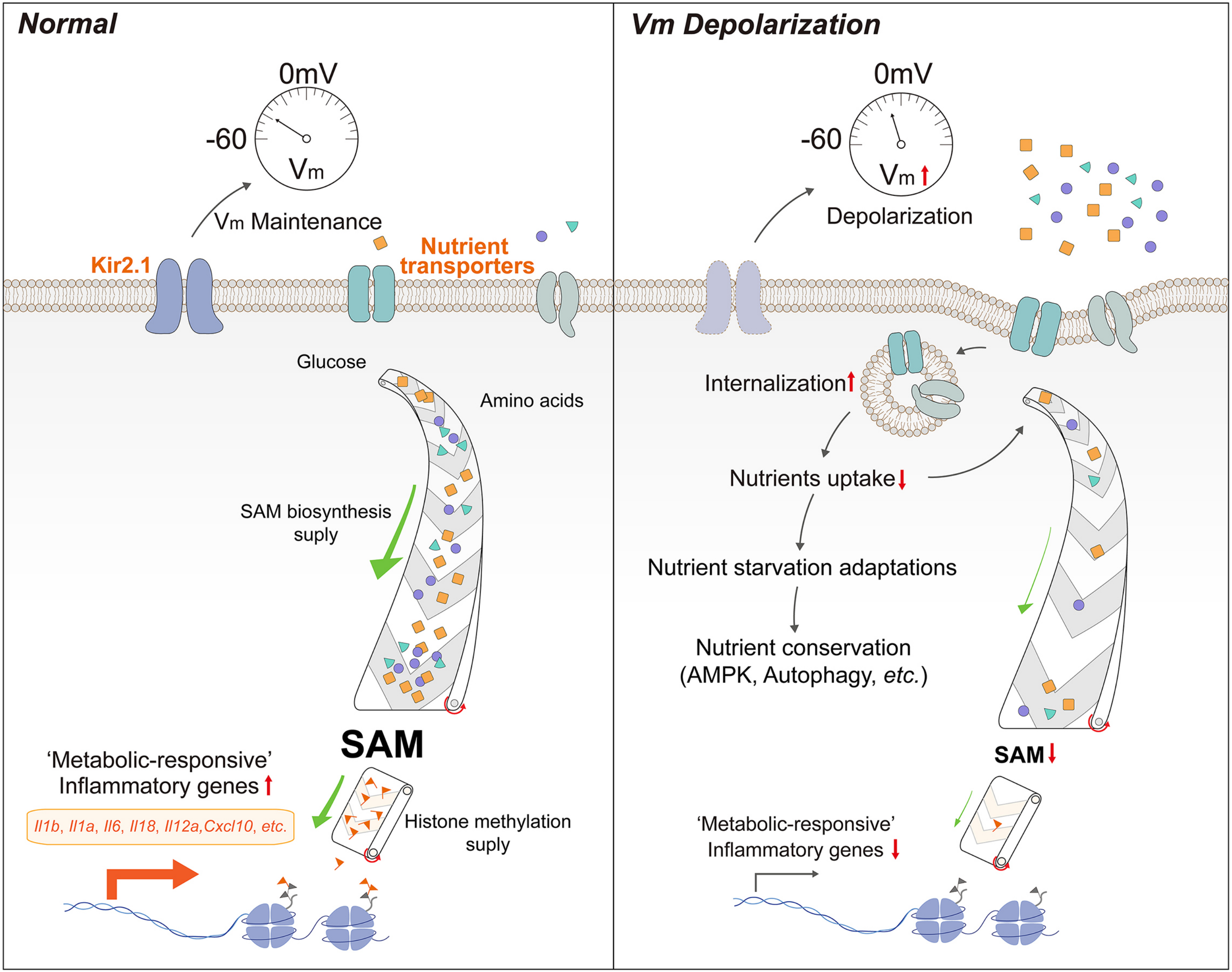
Normal
0mV
-60
Vm
Vm Maintenance
Kir2.1
Nutrient transporters
Glucose
Amino acids
SAM biosynthesis suply
SAM
'Metabolic-responsive' Inflammatory genes
Il1b, Il1a, Il6, Il18, Il12a,Cxcl10, etc.
Histone methylation suply
Vm Depolarization
0mV
-60
Vm
Depolarization
Internalization
Nutrients uptake
Nutrient starvation adaptations
Nutrient conservation (AMPK, Autophagy, etc.)
SAM
'Metabolic-responsive' Inflammatory genes

Supplementary Figure 11. Kir2.1-mediated macrophage V_m is a critical determinant of nutrient acquisition to feed the metabolic underpinnings of inflammation
