# Supplementary material for: Kir2.1-mediated membrane potential promotes nutrient acquisition and inflammation through regulation of nutrient transporters
Source: Nat Commun. 2022 Jun 21;13:3544. doi: 10.1038/s41467-022-31149-y (PMC9213538; doi:10.1038/s41467-022-31149-y)
Supplement: Supplementary file 3 — Reporting Summary [file 41467_2022_31149_MOESM3_ESM.pdf]

nature portfolio

Corresponding author(s): Di Wang

Last updated by author(s): Apr 4, 2022

Reporting Summary

Nature Portfolio wishes to improve the reproducibility of the work that we publish. This form provides structure for consistency and transparency in reporting. For further information on Nature Portfolio policies, see our Editorial Policies and the Editorial Policy Checklist.

Statistics

For all statistical analyses, confirm that the following items are present in the figure legend, table legend, main text, or Methods section.

n/a	Confirmed	
☐	☒	The exact sample size (*n*) for each experimental group/condition, given as a discrete number and unit of measurement
☐	☒	A statement on whether measurements were taken from distinct samples or whether the same sample was measured repeatedly
☐	☒	The statistical test(s) used AND whether they are one- or two-sided<br>*Only common tests should be described solely by name; describe more complex techniques in the Methods section.*
☒	☐	A description of all covariates tested
☐	☒	A description of any assumptions or corrections, such as tests of normality and adjustment for multiple comparisons
☐	☒	A full description of the statistical parameters including central tendency (e.g. means) or other basic estimates (e.g. regression coefficient) AND variation (e.g. standard deviation) or associated estimates of uncertainty (e.g. confidence intervals)
☐	☒	For null hypothesis testing, the test statistic (e.g. *F*, *t*, *r*) with confidence intervals, effect sizes, degrees of freedom and *P* value noted<br>*Give P values as exact values whenever suitable.*
☒	☐	For Bayesian analysis, information on the choice of priors and Markov chain Monte Carlo settings
☒	☐	For hierarchical and complex designs, identification of the appropriate level for tests and full reporting of outcomes
☐	☒	Estimates of effect sizes (e.g. Cohen's *d*, Pearson's *r*), indicating how they were calculated

Our web collection on statistics for biologists contains articles on many of the points above.

Software and code

Policy information about availability of computer code

Data collection	qPCR data was collected using Bio-Rad CFX Manager v3.1. Flow cytometry data was collected using ACEA NovoCyteTM (NovoExpress V1.5.0). Western Blot data was collected using ChemiScope3300 (ChemiCapture V2.5.5.103). Whole-cell current recordings were performed using a HEKA EPC10 amplifier controlled with PatchMaster software (HEKA epc10). Immunofluorescence Staining and Confocal Microscopy data was collected using a confocal microscope Zeiss LSM 800 (ZEN2 blue edition 2.0.0.0).
Data analysis	Flow cytometry was analyzed using FlowJo v10. Electrophysiology data was analyzed using Igor Pro 5.0. Figures were made using Graphpad Prism 9.1.0. For RNA-seq, pathway enrichment analysis of differentially-expressed genes (DEGs) was performed in Metascape (a gene annotation and analysis resource) and DAVID Bioinformatics Resources. Heatmap and bubble chart were made using RStudio1.1 or bioinformatics tools (hiplot.com.cn and www.bioinformatics.com.cn). The internalized ratio of Glut1 and 4F2hc were analyzed using ImageJ 1.53c. Membrane PIP2 fluorescence intensity were analyzed using ZEN2 blue edition 2.0.0.0. For membrane PIP2 fluorescence clustering analysis, Imaris 9.5.1 was used to quantity the number of PIP2 fluorescence spots. 3D reconstruction of membrane PIP2 was performed with Imaris 9.5.1. Metabolomic analysis was performed in MetaboAnalyst5.0, based on the KEGG and SMPDB databases.

For manuscripts utilizing custom algorithms or software that are central to the research but not yet described in published literature, software must be made available to editors and reviewers. We strongly encourage code deposition in a community repository (e.g. GitHub). See the Nature Portfolio guidelines for submitting code & software for further information.

Data

Policy information about availability of data

All manuscripts must include a data availability statement. This statement should provide the following information, where applicable:

- Accession codes, unique identifiers, or web links for publicly available datasets
- A description of any restrictions on data availability
- For clinical datasets or third party data, please ensure that the statement adheres to our policy

The RNA-seq data in this paper have been deposited in the Gene Expression Omnibus (GEO) with accession number GEO: GSE146158, GSE183067.

Field-specific reporting

Please select the one below that is the best fit for your research. If you are not sure, read the appropriate sections before making your selection.

☒ Life sciences ☐ Behavioural & social sciences ☐ Ecological, evolutionary & environmental sciences

For a reference copy of the document with all sections, see nature.com/documents/nr-reporting-summary-flat.pdf

Life sciences study design

All studies must disclose on these points even when the disclosure is negative.

Sample size	No sample-size calculations were performed. Sample sizes were determined by magnitude and consistency of measurable differences.
Data exclusions	For cell experiments, none of the samples was excluded. For animal experiments, none of the animals was excluded from the analysis except for animals that were dead or no enough sample were collected.
Replication	The Western Blot and q-PCR results are representative of at least three independent successfully repeated experiments to ensure the data reproductivity
Randomization	Within experiments, mice were littermates, sex-matched, weight-matched and age-matched. Wild type mice were randomly allocated to different groups and received different treatments.
Blinding	No blinded procedure needed during experiments, for all samples were conducted at the same condition and data were collected in the same instrucments for all experiments and there are no experiments that require scoring by subjective judgment.

Reporting for specific materials, systems and methods

We require information from authors about some types of materials, experimental systems and methods used in many studies. Here, indicate whether each material, system or method listed is relevant to your study. If you are not sure if a list item applies to your research, read the appropriate section before selecting a response.

Materials & experimental systems

n/a	Involved in the study	
☐	☒	Antibodies
☐	☒	Eukaryotic cell lines
☒	☐	Palaeontology and archaeology
☐	☒	Animals and other organisms
☐	☒	Human research participants
☒	☐	Clinical data
☒	☐	Dual use research of concern

Methods

n/a	Involved in the study	
☒	☐	ChIP-seq
☐	☒	Flow cytometry
☒	☐	MRI-based neuroimaging

Antibodies

Antibodies used	The following antibodies were used:pro-IL-1β (AF-401-NA, 1:1000, WB) was from R&D systems ; anti-Kir2.1 (19965-1-AP, 1:1000, WB) was from Proteintech; anti-phospho-p65(#3033, 1:1000, WB), anti-p65(#8242, 1:1000, WB), anti-phospho-IκBα(#2859, 1:1000, WB), anti-IκBα(#4814, 1:1000, WB), anti-phospho-Erk(#4370, 1:1000, WB), anti-Erk(#4695, 1:1000, WB), anti-phospho-JNK(#9255, 1:1000, WB), anti-JNK(#9252, 1:1000, WB), anti-phospho-P38(#4511, 1:1000, WB), anti-P38(#8690, 1:1000, WB), anti-phospho-AMPK (#2535, 1:1000, WB), anti- AMPK (#5831, 1:1000, WB), anti-H3K36me3 (#4909, 1:200 CHIP) all from Cell Signaling Technology; anti-LC3B (A19665, 1:1000, WB) was from ABclonal; anti-GLUT1 (ab115730,1:1000 for WB, 1:200 for flow cytometry and immunofluorescence), mouse monoclonal [2C11] to PIP2 (ab11039, 1:200, immunofluorescence), goat polyclonal secondary antibody to mouse IgG - H&L (Alexa Fluor® 647) (ab150115, 1:200, immunofluorescence) all from Abcam; PE anti-mouse 4F2hc (#128207, 1:200, flow cytometry), APC/Cyanine7 anti-mouse CD45 (103115, 1:100, Flow Cytometry), PE/Cyanine7 anti-mouse/

	human CD11b (101215 , 1:100, Flow Cytometry), APC anti-mouse F4/80 Antibody (123115 , 1:100, Flow Cytometry), APC anti-mouse Ly-6C Antibody (128015, 1:100, Flow Cytometry) was from Biolegend; anti-4F2hc (15193-1-AP, 1:200, immunofluorescence) was from Proteintech; anti-β-actin (M1210-2, 1:3000, WB), anti-rabbit IgG-HRP (HA1001, 1:3000, WB), anti-mouse IgG-HRP (HA1006, 1:3000, WB), and anti-goat IgG-HRP (HA1005, 1:3000, WB) all from HuaBio; DyLight549 goat anti-rabbit IgG [H+L] (70-GAR5492), DyLight488 goat anti-rabbit IgG [H+L] (70-GAR4882) all from MultiSciences; ChIP kit (#9005) was from Cell Signaling Technology; ELISA kit for IL-1β (#88-8014-22) and IL-6 (#88-7064-88) were from ThermoFisher; ELISA kit for IL-1α was from DAKEWE (1210112); and PI(4,5)P2 Mass ELISA kit was from Echelon (K-4500).
Validation	Antibodies and Kits were used according to the manufacturer's guideline. The Research Resource Identifier(RRID) numbers for antibodies and Kits are as follows: pro-IL-1β (AF-401-NA, AB_4166840), anti-Kir2.1 (19965-1-AP, AB_10859827), anti-phospho-p65 (#3033, AB_331284), anti-p65(#8242, AB_10859369), anti-phospho-IκBα(#2859, AB_561111), anti-IκBα(#4814, AB_390781),nti-phospho-Erk(#4370, AB_2315112), anti-Erk(#4695, AB_390779), anti-phospho-JNK(#9255, AB_2307321), anti-JNK(#9252, AB_2250373), anti-phospho-P38(#4511, AB_2139682), anti-P38(#8690, AB_10999090), anti-phospho-AMPK (#2535, AB_331250), anti- AMPK (#5831, AB_10622186), anti-H3K36me3 (#4909, AB_1950412), anti-LC3B (A19665, AB_2862723), anti-GLUT1 (ab115730, AB_10903230), mouse monoclonal [2C11] to PIP2 (ab11039, AB_442848), goat polyclonal secondary antibody to mouse IgG - H&L (Alexa Fluor 647, ab150115, AB_2687948), PE anti-mouse 4F2hc (#128207, AB_1186107), APC/Cyanine7 anti-mouse CD45 (103115, AB_312980), PE/Cyanine7 anti-mouse/human CD11b (101215, AB_312798), APC anti-mouse F4/80 Antibody (123115, AB_893493), APC anti-mouse Ly-6C Antibody (128015, AB_1732087), anti-4F2hc (15193-1-AP, AB_2254909), anti-rabbit IgG-HRP (HA1001, AB_2819166), anti-mouse IgG-HRP (HA1006, AB_2819167), ELISA kit for IL-1β (88-8014-22, AB_2575169), ELISA kit for IL-6 (88-7064-88, AB_2574990). For PIP2 immunofluorescence, antidody was used with some modification described in manuscript .Cells were rapidly fixed by 4% FA and 0.2% GA (glutaraldehyde) in PBS. Fixation was allowed to proceed for 15 min at room temperature (20–24 ◦C). Then cells were rinsed three times with PBS containing 50 mM NH4Cl. Slides were then placed on a metal plate in a deep ice bath and chilled for at least 2 min. All subsequent steps were performed on ice, with all solutions pre-chilled. After that, cells were blocked and permeabilized for 45 min with a solution of buffer A (20 mM Pipes, pH 6.8, 137 mM NaCl, 2.7 mM KCl) containing 5% (v/v) NGS (normal goat serum), 50 mM NH4Cl and 0.5% saponin. Primary antibodies were applied in buffer A with 5% NGS and 0.1% saponin for1 h. After two washes in buffer A, a 45 min incubation with secondary antibody in buffer A with 5% NGS and 0.1% saponin was performed. Slides were then rinsed four times with buffer A, and cells were post-fixed in 2% FA in PBS for 10 min on ice, before warming to room temperature for an additional 5 min. FA was removed by three rinses in PBS containing 50 mM NH4Cl, followed by one rinse in distilled water. Cells were then stained with DAPI (4,6-diamidino-2-phenylindole) and covered with glass cover slips, and sealed with nail varnish.

Eukaryotic cell lines

Policy information about cell lines

Cell line source(s)	HEK293T and THP-1 (ATCC), iBMDM (Feng Shao ,National Institute of Biological Sciences, China)
Authentication	cell lines were authenticated by STR profiling.
Mycoplasma contamination	Cell lines were tested negative for mycoplasma
Commonly misidentified lines (See ICLAC register)	No commonly misidentified cell lines were used

Animals and other organisms

Policy information about studies involving animals; ARRIVE guidelines recommended for reporting animal research

Laboratory animals	Mice, strain C57Bl/6, Lyz2-cre, kcnj2f/f mice , males and females, 6-12 weeks of age. Animals were housed in a specific pathogen-free facility maintained below 22 °C and 55% humidity under a 12-h light-dark cycle and free access to food and water in the University Laboratory Animal Center
Wild animals	None
Field-collected samples	None
Ethics oversight	All of the animal experimental protocols in this manuscript were approved by the Review Committee of Zhejiang University School of Medicine and were in compliance with institutional guidelines.

Note that full information on the approval of the study protocol must also be provided in the manuscript.

Human research participants

Policy information about studies involving human research participants

Population characteristics	Synovial fluid (approximate 4-5 ml) was obtained from two gouty patients (a 36-year-old man and a 56-year-old man) with serum uric acid levels > 500 mmol/l and knee effusion. The patients were not involved in previous procedures or drug tests.
Recruitment	Gouty patients were recruited in Department of Rheumatology of the Second Affiliated Hospital of Zhejiang University School of Medicine. Gouty patients (a 36-year-old man and a 56-year-old man) with serum uric acid levels >500 mmol/l and knee effusion. The patients were not involved in previous procedures or drug tests, for this may affect the inflammatory response of Synovial fluid cells in the in vitro experiments.
Ethics oversight	To use these clinical materials for research purposes, prior patient written informed consent and approval from the

Institutional Research Ethics Committee of The Second Affiliated Hospital of Zhejiang University School of Medicine were obtained (approval no. 2018-064).

Note that full information on the approval of the study protocol must also be provided in the manuscript.

Flow Cytometry

Plots

Confirm that:

- [x] The axis labels state the marker and fluorochrome used (e.g. CD4-FITC).
- [x] The axis scales are clearly visible. Include numbers along axes only for bottom left plot of group (a 'group' is an analysis of identical markers).
- [x] All plots are contour plots with outliers or pseudocolor plots.
- [x] A numerical value for number of cells or percentage (with statistics) is provided.

Methodology

Sample preparation	For peritoneal macrophages(PMs), mice are sacrificed 3-5 days after i.p. injection of thioglycolate and the peritoneal exudate cells are collected. Mouse peritoneal macrophages (6×10^5 cells) were seeded and stimulated under the indicated conditions. Then the cells were collected with a cell scraper, followed by antibody staining for 30 min at 4**℃.** After washing with PBS to exclude non-specific staining, the surface expression of CD98 or GLUT1 was detected by flow cytometry (ACEA NovoCyte). For in vivo glucose uptake assays, 8-week-old mice were intraperitoneally injected with LPS (1 mg/kg); 30 min later 2NBDG (500 nmol/mouse) was injected, and after another 1 h, blood and peritoneal exudate cells were sequentially collected. Peripheral blood mononuclear cells were isolated from blood samples using Ficoll-PaqueTM PLUS (GE Healthcare) according to the manufacturer's instructions. Peritoneal exudate cells (PECs) were flushed with 5ml cold PBS. Macrophages (CD45+ CD11b+ F4/80+ cells) in PECs and monocytes (CD45+ CD11b+ Ly6Chigh cells) in the blood were stained with the corresponding antibody for 30 min on ice. After washing in PBS, FACS analysis of 2NBDG MFI was carried out with a BD Fortessa Multicolor flow cytometer.
Instrument	ACEA NovoCyteTM
Software	ACEA NovoExpress are used for collecting flow cytometry raw data and statistics are analyzed in Flowjo X.
Cell population abundance	For in vitro flow cytometry experiments, cell population numbers are almost 3-6×10^5 per sample, more than 95% cells are macrophages and about 30-50% cells are alive verified by FVD staining.
Gating strategy	After FSC/SSC starting gate, cells are sequential analyzed FSC-A/FSC-H to remove adhesive cell mass and FVD to remove dead cells. F4/80+ cells are finally picked for glucose uptake analysis, intracellular K+ detection and surface marker indication, respectively.

- [x] Tick this box to confirm that a figure exemplifying the gating strategy is provided in the Supplementary Information.
